# The foreign language effects on strategic behavior games

**DOI:** 10.1371/journal.pone.0277556

**Published:** 2022-11-17

**Authors:** Zilu Wang, Michael C. W. Yip

**Affiliations:** Department of Psychology, The Education University of Hong Kong, Ting Kok, Hong Kong; Zhejiang University of Finance and Economics, CHINA

## Abstract

The present study examined foreign language effects on the decisions made in a series of strategic behavioral games (e.g., the Prisoner’s Dilemma, the Oligopolistic Competition, and the Volunteer’s Dilemma). We recruited 154 native Chinese-speaking university students, with English as their second language, as participants. They were asked to make decisions while playing four simple behavioral games in either Chinese or English language version and to complete a Language History Questionnaire. The results showed that 1) the participants in each language group performed differently in the Prisoner’s Dilemma Game and in one condition of the Volunteer’s Dilemma Game which involved a relatively high level of uncertainty; and 2) foreign language proficiency, frequency of application and cultural identity triggered by the corresponding foreign language moderated the foreign language effects. This pattern of results is consistent with the Cultural Accommodation Hypothesis and the risk-aversion preference to use one’s native language.

## Introduction

One basic function of language is information transmission, and this transmission can influence various aspects of our cognitive processes, including decision making. Since the last century, researchers have analyzed how language impacts on our decision-making, that is framing effects. Tversky and Kahneman [1] recruited two groups of participants to make choices regarding a common situation (The Asian Disease Paradigm). These choices were described as either "gain-framed" or “loss-framed” policies. The authors observed that when a case was phrased in terms of potential gains, people tended to choose the less risky option. Conversely, when the participants confronted a potential loss scenario, they gravitated towards the option that required greater risk. This experiment confirmed that language-framed statements (or problems) can clearly influence people’s decision-making processes. Over the decades, this theory has been replicated and accepted well by specialists from various fields, including but not limited to behavioral economists, linguists, and psychologists [2]. Although the study of framing effects generated by descriptive language has been investigated many times, it was only in the past few years that scholars started to analyze how the ‘nativeness’ of a language influences decisions [3].

More than half of the world’s population nowadays is capable of communicating in a foreign language [4], and this number has increased steadily in recent years. Along with the trend of globalization, English serves as the lingua franca that builds connections among speakers with different native tongues and diverse cultural backgrounds [5]. Early in the 21^st^ century, three quarters of English users were non-native speakers who learned it as a foreign language in classroom settings [6]. Due to this large population of foreign language users, researchers have recognized that it is vital to learn how and why the use of a foreign language influences an individual’s choices in different situations [7].

Numerous studies have shown that thinking in a foreign language can lead to both positive and negative decision-making processes and outcomes. On one hand, some scholars have stated that using a foreign language can reduce biases in decision-making, because this process enables people to rely on systematic processes that are more remote from the immediate intuitive system than they are when one is thinking in one’s native language [8]. For example, when using their native language, one study’s participants were less likely to overestimate the likelihood of positive outcomes after a series of previous successes than when the situations were presented in their second language [8]. In addition, Keysar, Hayakawa and An [9] reported that, when the Asian Disease Paradigm was introduced in a foreign language, asymmetry in framing effects was lessened in relation to both losses and gains. On the other hand, some researchers have also suggested that a foreign language may interfere with one’s train of thoughts from multiple perspectives. At first, performance may be limited by foreign language anxiety, especially during listening and speaking sessions. The anxious states of foreign language users can reduce the accuracy of both information input and output [10]. Also, generating messages in a non-native tongue may induce a greater burden on the cognitive load and hence reduce the efficiency of decision-making [11]. De-contextualization can also be triggered because each language requires a specific cultural background that is quite challenging for language learners to understand fully [12].

Various aspects of the effects of thinking in a foreign language have been investigated. These have included role inferences [13], moral judgements [14, 15], and affective processing [14]. These studies focused on the outcomes of high-level cognitive processes like reasoning, perception and thinking. Hence, the procedures used in these studies have always consisted of continuous responses from participants in multiple trials with scenarios that are relatively rare in daily life.

To investigate further how the use of a foreign language influences regular human performance in day-to-day living, an analysis of other daily higher-order cognitive processes may enrich understanding about the underlying psychological mechanisms and the linkage between foreign languages and human cognition [16]. This line of research should use simple designs and concrete contexts as the testing materials [17]. Game Theory may serve as one compatible and appropriate tool for studying the fundamental processes involved in strategic decision-making process. Therefore, the present study used several simple Game Theory scenarios that usually occur in real-life business settings to look into the relationship between the effects of foreign language and human cognition (in particular, our decision-making processes).

In this research area, previous experiments mostly investigated cases within Anglophone languages (e.g., English), Latin European Languages (e.g., Spanish, Italian and French) or Germanic European languages (e.g., Dutch and German). These languages belong to the Indo-European Language systems, and they share a certain degree of intercultural similarities [18]. Studies of foreign language effects on individual responses have rarely been conducted between Chinese and English languages [19]. Compared to all the languages used in past research endeavors, Chinese and English languages share comparatively few intercultural similarities. Therefore, the present study aimed to investigate the foreign language effects on decision-making from this cross-cultural perspective.

Based on Boroditsky’s studies is the view that thinking in one language leads to culture-based decisions [20, 21]. According to Boroditsky [21], different languages build up various meaning-generating frameworks due both to the rules of expression (grammar, words) and language experiences (patterns and values). Cultural accommodation has been applied widely to identify and explain the underlying mechanisms of foreign language effects [22]. When processing occurs in a certain language, cultural constructs of that language are activated. Harzing [23] invited participants from 24 different countries to respond to a questionnaire. Two experimental groups of non-native English speakers were established. In one of these groups, the participants were asked to provide responses in English, while those in the other group were required to respond in their native tongue, while the control group consisted of native English speakers. The results showed that cultural differences among regions were reduced when the participants responded to the questions in the language in which they were presented. Previous literature suggested that cultural accommodation may start from incidental acculturation [24]. Studying a new language may guide the new language learners to know about the corresponding culture. Norms and values of that culture eventually influence a learner’s perceptions. This results in modified behaviors influenced by language [25].

How do we classify the underlying cultures of different regions? Researchers have suggested numerous systems to induce unique characteristics. Cattell [26] concluded that patterns of culture emerged from the syntax dimensions. Hofstede [27] highlighted espoused values of individuals from various nationalities, and designed a classification system by assigning countries into cultural clusters based on four dimensions of variation: Power Distance, Uncertainty Avoidance, Individualism and Masculinity [28]. One of the most globalized classifications used currently was developed by the GLOBE project, which included 61 nations [29]. In this project, the researchers enhanced the original four dimensions proposed by Hofestede [30] by separating social practices and social values. This permitted the independent analysis of the influences of social values on individuals [31]. Here, because we aimed to study foreign language effects on individuals’ responses to strategic games (Cooperative vs. Competing, Volunteering vs. Selfish), the classification model proposed by the GLOBE project was most appropriate.

According to the GLOBE Project’s Societal Cluster classification model [29], the Chinese language belongs to the Confucian Asia Cluster, while the English language belongs to the Anglo Cultural Cluster. Compared to the Anglo Cultural Cluster, Confucian Asians are characterized by higher values in terms of both institutional and in-group collectivism, higher levels of uncertainty avoidance and more support towards masculinity in their mindsets [32]. People from the Anglo Cultures Cluster tend to be more agreeable to individualism and gender egalitarianism, but they are less likely to exhibit uncertainty avoidance [33]. When applying culture patterns to the analysis of individuals’ strategic behaviors, several assumptions emerge. First, because of the higher tendency towards collectivism, people immersed in Confucian Asian cultures may tend to play cooperative rather than competing roles. Second, due to their higher uncertainty avoidance, Confucian Asians may be less likely to cooperate when the costs of the cooperation are not clear. Therefore, when language contexts activate cultural priming, behavioral tendencies occur [34]. Related to the idea of cultural accommodation, the first hypothesis (H1) was about participant tendencies displayed when playing cooperative roles in the games in different languages. We predicted that in the Prisoner’s Dilemmas, Chinese natives would be less likely to cooperate when the contexts were displayed in English; while in the Volunteer’s Dilemmas, Chinese native speakers would be less likely to cooperate when the contexts were described in their native language.

In addition, past studies have indicated that foreign language processing does not influence people in the same ways, even if they share the same native tongue. These differences could be explained by several language-related factors. First, Volk and his colleagues [11] indicated that the increased cognitive loads in foreign language processing bounded the rationality of a human’s decisions. However, this was less likely to occur in the more proficient foreign language users [35]. Also, language users’ self-evaluations of their proficiency may be negatively related to the level of foreign language anxiety, which might lead to biases and misjudgments in decision making. Since foreign language proficiency can improve cognitive fluency, it could be a moderator of foreign language effects.

Second, a foreign language can increase the psychological distance, which can influence people to rely more on their general and fundamental attitudes [36]. More frequent use of the foreign language could reduce the psychological distance by involving more direct experiences in the foreign language processing [37]. Hence, frequency of use of the foreign language might influence the effects of foreign language processing.

Furthermore, as language priming leads people to accommodate the norms and values corresponding to that language, personal connection and agreeableness with the culture are related to the tendency to display cultural-based behaviors [34]. Hence, we assumed that cultural identity with the corresponding language could have an impact on the outcomes of foreign language priming. Therefore, to investigate the influences of the three factors discussed above, the second hypothesis (H2) focused on the correlations between the scale of foreign language effects and these three factors. We predicted that foreign language proficiency, frequency of use of the foreign language and cultural identity with the corresponding language could be correlated to the strength of the foreign language effects.

## Method

### Participants

We recruited 154 University students (Male: 41 and Female: 113, mean age: 22 years) from Confucian Asia Cluster regions, China, Hong Kong, Macau, and Taiwan. Anyone with prior exposure to related game-theory concepts (i.e., had studied related courses or participated in similar games) was excluded from the sample. The participants were required to complete a Language History Questionnaire, LHQ2.0 [38] to record their demographical information, language experiences and language proficiency. All participants were native Chinese speakers who had been learning English as a foreign language for more than 10 years. They used both Chinese and English in their daily lives. Written consent was obtained from all participants before the start of the study, and they were all informed verbally about the procedure of the experiment. This study was approved by the HREC of the Education University of Hong Kong and all methods and procedures were carried out according to the guidelines and regulations approved by the University.

### Materials

Four classical behavioral games were used in the present study (S1 Appendix). These were the Prisoner’s Dilemma, the Oligopolistic Competition (an application of the Prisoner’s Dilemma) and two different situations of the Volunteer’s Dilemma with varying degrees of personal loss in volunteering [39].

Gaming provides straightforward and concrete models to study the fundamental processes of human behavior [40]. The strategic elements underpinning the game settings allow players to generate information while planning and behaving strategically [41]. Players’ strategic responses not only indicate their perceptions and thinking processes, but also provoke real decisions with corresponding payoffs. Their actual behaviors serve as accessible forms of evidence that are suitable for analyzing their preferences and the rationalities of their choices [42]. However, sometimes their responses could deviate from rational assumptions. The failures of participants to maximize their payoffs could encourage researchers to discover alternative explanations for the bounded rationality by reflecting the fundamental processes. Modeling the players’ performances in experimental behavior games involves the strict control of variables under the standard structures [43].

Furthermore, players’ responses to the games could also reflect their decisions in real-life social contexts under similar situations [44]. Behavioral games are derived from social interactions and personal conflicts. Therefore, the behavioral patterns demonstrated in games are aligned with the players’ corresponding field performances [45].

#### The Prisoner’s Dilemma and the Oligopolistic Competition

The applications of the Prisoner’s Dilemma have been applied widely in evaluating the strategic behaviors of participants in terms of cooperation vs competition [46].

The Prisoner’s Dilemma game (Game 1) is one of the classic and ultimate experimental games which has been applied to various fields of social sciences. It is a simple matrix game that involves two parties under the same trial. Each player has two choices, cooperating by confessing or defecting by denying the crime that they committed together. In order to maximize the payoff, the Nash Equilibrium would suggest the player should cooperate rather than defect regardless of the response of the other [45].

The Oligopolistic Competition (Game 2) is an application of the Prisoner’s Dilemma in business contexts. In this game, two firms from the same industry have to decide either to promote themselves or not. The increased popularity could increase the firm’s market share. In order to maximize the payoff, the Nash Equilibrium would recommend the player to place an advertisement rather than to do nothing, regardless of the other’s player’s response. Unlike the original Prisoner’s Dilemma, a competing choice would lead to a more substantial payoff in this situation [45].

The English versions of these two games were adapted from Weibull [39]. Details of each game were paraphrased due to the reported complexity from pilot trials. The Chinese version was first translated by a qualified translator with the CATTI-II Certificate and then reviewed by one of the authors (a fluent bilingual psycholinguist) and two research assistants. During the translation process, an effort was made to ensure the appropriateness of the language and the cultural context in order to maintain the content validity. The Chinese version was again back translated into English by a professional bilingual editor. The authors again reviewed the resultant English back-translation version. All of the final modifications received unanimous agreement from the whole research group.

#### The Volunteer’s Dilemmas

The responses from players in the Volunteer’s Dilemmas reflected their perceptions of uncertainty, including attitudes towards potential risks and losses. When the potential costs of volunteering differed, the possibility of participants cooperating (volunteering) were differentiated [47].

In Volunteer’s Dilemmas, if (at least) one participant volunteered himself or herself with a personal cost, the others would benefit from this public good. If other players had already volunteered, then the cost of volunteering would be wasted. However, if nobody decided to be the volunteer, the whole group would receive no payoff [44].

In this case, we applied the two conditions of the Volunteer’s Dilemma which required participants to pay different levels of cost. The first one (Game 3) described a blackout in a community. They needed a volunteer to call the administrators of a power supply to restore it. The last game (Game 4) required a higher cost from the volunteer. It constructed an emergency landing on a desert land, with a volunteer needed to swim to a nearby inland in order to send out an SOS message. Apart from the physical output, the cost also accounted for the danger associated with the uncertainty.

The English version of these two games was adapted from Poundstone [48]. The same translation and back-translation procedures described for the first two games were used for the Chinese version.

### Procedure

The participants were assigned randomly into one of two language groups (either Chinese or English). All the information provided (i.e., information sheet, consent form, instructions and games) were identical except for the languages used. In the experiment, the participants were first invited to give their responses to a series of four strategic behavioral games [the Prisoner’s Dilemma, the Oligopolistic Competition (an application of the Prisoner’s Dilemma) and two conditions of the Volunteer’s Dilemma that required various degrees of personal losses in volunteering]. Binary choices (A or B) were provided for each situation. The participants were told that they could choose either A or B (i.e., cooperate or compete) under each condition. It was optional for them to write down the reasons for their decisions. To prevent the activation of other higher-order cognitive processes like calculating, the game was presented in a descriptive language without displaying any forms or charts about the payoffs following each choice. All the participants completed each game individually, using fictitious player(s). No immediate feedback was provided after the participants had made their decisions. Immediately after the experiment, they were required to complete the LHQ2.0 to record other language-related variables. The whole study lasted about 10 minutes.

## Results

**Fig 1:**
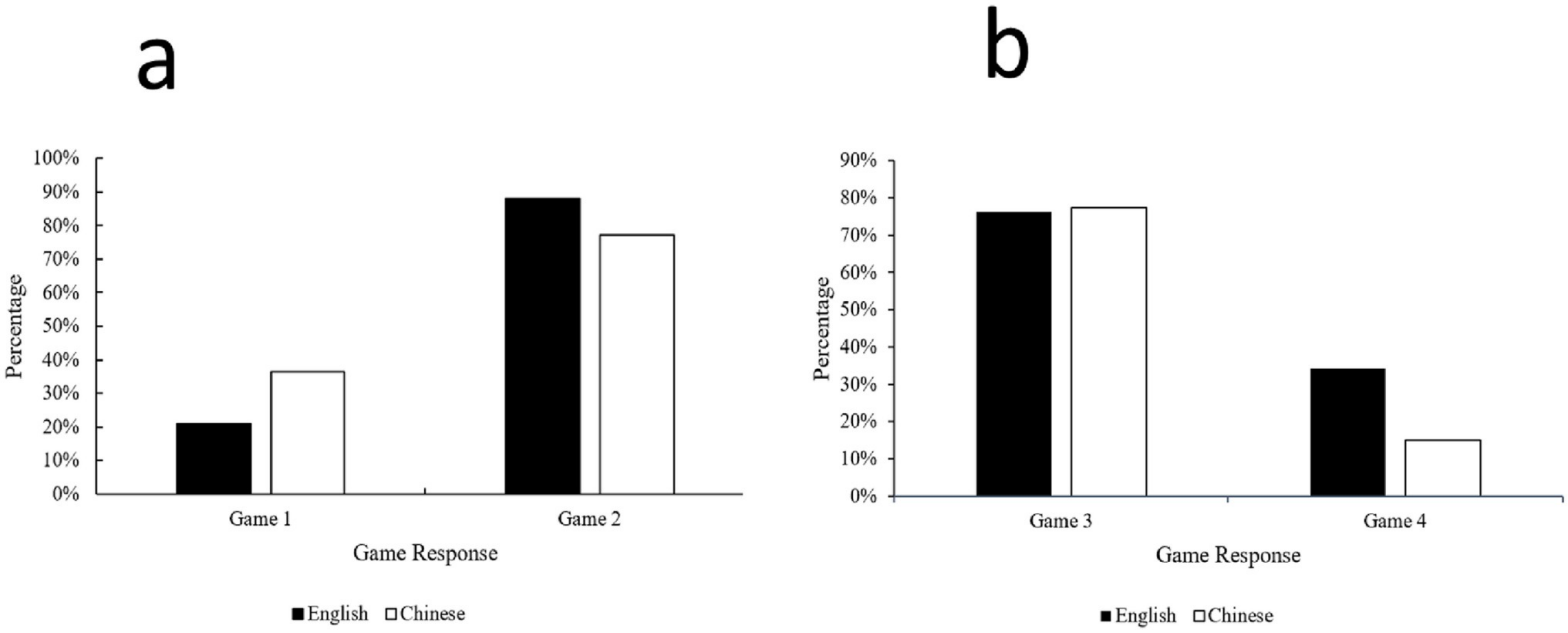
The percentages (%) of cooperation for the four games in each language group. a: Percentage of cooperation in Prisoner’s Dilemmas; b: Percentage of cooperation in Volunteer’s Dilemmas.

The main dependent variable in each trial was the binary choice between cooperating and competing. We coded the choice of “cooperating” as value 1 while the choice of “competing” as value 0. First, we conducted Chi-square tests of independence (χ) in each round to examine the association between the types of language and likelihood of cooperating (H1). Second, we used correlational analyses of the participant responses and other variables, namely the participants’ foreign language proficiency, frequency of foreign language use and cultural identity with the corresponding foreign language to examine the other hypothesis (H2). The percentages (%) of cooperation for the four games in each language group are presented in Fig 1. The correlation matrix of those language-related variables is presented in Table 1.

**Table 1:** Correlations of the language-related factors when the contexts were presented in the foreign language.

Factor	1	2	3
Game			
1	-.382**	-.453**	-.462**
2	.211	.385**	.362*
3	.185	.253	.387**
4	.655**	.723**	.717**

Note^1^: Factor 1 = Language proficiency, Factor 2 = Frequency of use of the foreign language, Factor 3 = Cultural identity.

Note^2^:

* *p* < .05;

** *p* < .01.

### Results for Game 1

There was a significant association between the language and likelihood of cooperating, with χ^2^ (1, *N* = 154) = 4.258, *p* = .046. The participants in the foreign language group were observed as less likely to cooperate (21% vs. 36%). When the contexts were presented in the foreign language, the likelihood of cooperating and all the participants’ variables (foreign language proficiency [r(77) = -.382, *p* < .01], frequency of foreign language use [*r*(77) = -.453, *p* < .01] and cultural identity to the corresponding foreign language [*r*(77) = -.462, *p* < .01]) were negatively correlated. The results supported that the more proficient of the foreign language and frequently use of the foreign language of the participants, the stronger the foreign language effects for them in making descsion.

### Results for Game 2

The association between language and the participants’ responses was not significant, χ^2^ (1, *N* = 154) = 2.535, *p* > .05, with only a 7% difference. When the contexts were presented in the foreign language, the likelihood of cooperating and the participants’ variables (frequency of foreign language use [*r*(77) = .385, *p* < .01] and cultural identity with the corresponding foreign language [*r*(77) = .362, *p* < .05]) were positively correlated. However, the variable “foreign language proficiency” was not significant statistically [*r*(77) = .211, p = n.s.] though in the expected poisitve direction.

### Results for Game 3

The association between language and the possibilities of volunteering (cooperating) was not significant, χ^2^ (1, *N* = 154) = .024, *p* > .05, with only a 1% difference. When the contexts were presented in the foreign language, the possibilities of volunteering and the participants’ cultural identity with the corresponding foreign language were positively correlated (*r*(77) = .387, *p* < .01). However, the other two language-related variable “foreign language proficiency” [*r*(77) = .185, *p* = n.s.] and “frequency of foreign language use” [*r*(77) = .253, *p* = n.s.] were not significant statistically.

### Results for Game 4

There was a significant association between language and the possibilities of volunteering (cooperating), with χ^2^ (1, *N* = 154) = 6.951, *p* = .010. The participants in the foreign language group were more likely to volunteer themselves (34% vs. 14%). When the contexts were presented in the foreign language, the likelihood of cooperating and all the participants’ variables (foreign language proficiency [r(77) = .655, *p* < .01], frequency of foreign language use [*r*(77) = .723, *p* < .01] and cultural identity with the corresponding foreign language [*r*(77) = .717, *p* < .01]) were positively correlated. Similar to Game 1, the pattern of results provided further support to the foreign lanaguge effects for the participants to make a decision.

In a summary, the first hypothesis (participants’ behavioral tendencies to cooperate would be different when they were making decision in different languages) was supported by the results for Game 1 and Game 4 but not by those for Game 2 or Game 3. The second hypthesis was also supported by the results of Game 1 and Game 4, but not by those for Game 2 and Game 3 (i.e., not all participants’ langauge-related variables were correlated). That is, the foreign language proficiency, frequency of foreign language use and the cultural identity to the corresponding foreign language were, to an extent, associated with the intensity of the foreign language effects. Together, the pattern of results supported the cultural accommodation explanation to participants’ behaviors tendencies when the decision scenarios were presented in a foreign (or second) language context.

## Discussion

Previous studies have demonstrated clearly that language affects the way we think, and recent studies have also reported that our (cognitive) processing in a foreign language not only alters our attitudes and perceptions, but also impacts actual behaviors [13]. Here, we extended these observations to another important cognitive process, decision making. In the present study, we used Chinese and English as the focus languages to investigate the foreign language effects on human decision making while playing strategic games. Past relevant research on how foreign language affects cognitive processing focused on a few higher-order thinking processes, such as moral judgment [49], reasoning [50] or time-perception [51]. This was one of the first experimental studies to use Chinese-English bilinguals in research of this nature. In general, the overall patterns of results were in line with the cultural accommodation hypothesis [22] as well as the risk-aversion preference when using one’s native tongue [9].

Our results from games 1 and 4 supported the main hypothesis about foreign language effects. In the Prisoner’s Dilemma Game, when the contexts were described in Chinese (their native tongue), the participants were more likely to provide cooperative responses than when the contexts were presented in the foreign language. First, the participants’ behavior patterns were in accord with the norms and values of the culture backgrounds corresponding to each language [52]. This supported the assumption of cultural accommodation. Language is a valid source of cultural priming since each language is associated with a specific background. Thus, when contexts presented in different languages activate cultural priming, behavioral tendencies will occur [53]. The negative correlation between cultural identity with the corresponding foreign language and the likelihood of cooperation could also be explained by the Cultural Accommodation Hypothesis, as the agreeableness and connection to cultures corresponding to the foreign language enlarged the performance of language-specific responses. Second, regarding the reduction in emotional resonance of processing in a foreign language [54], when the game was presented in English, the participants (or players) might have perceived a lower degree of risks in competing. On the opposite side, those who went through this game in their native language might have felt a relatively higher level of risks for defecting [35]. Therefore, the risk-aversion tendency in the native language group increased the possibility of cooperative behaviors.

From the outcomes of each choice, cooperating was the main strategy that maximized the respondent’s personal gains in this game [39]. Lower rates of cooperation in the English language group showed that decision-making bias was increased by foreign language processing. Volk et al. [11] suggested that foreign language processing could induce heuristic bias in decision making, which is consistent with the present results. However, the foreign language effects are not always negative. Keysar, Hayakawa and An [9] argued that if decisions require less emotional reactions, foreign language(s) could depend more on systematic processes, thereby reducing bias. Therefore, the consequences of foreign language effects are influenced by the requirements of each task.

Similarly, in the Volunteer’s Dilemma Games, when the cost of cooperating (volunteering) involved higher levels of uncertainty (in Game 4), the participants were more likely to give cooperative responses if the contexts were described in English (the foreign language). The explanations for this are similar to those for the PD (Prisoner’s Dilemma) games. According to the Cultural Accommodation theory, the Chinese language primes the values of the Confucian Asian cultures. As Confucian Asians are typically less like to cooperate when a situation involves uncertainty, the rate of volunteering was relatively lower in this study when the contexts were presented in the Chinese language. Similarly, the higher acceptance of uncertainty triggered by the English language increased the possibilities of volunteering in this case [19]. The positive correlation between cultural identity with the responding foreign language and likelihood of cooperating could also be explained by the Cultural Accommodation Hypothesis, as the agreeableness and connection to the culture corresponding to the foreign language enlarged the performance of language-specific responses [55].

Second, regarding the increase of psychological distance in processing in a foreign language [37], when the game was presented in English, the players might have perceived lower degrees of loss in volunteering. On the opposite side, those who went through this game in their native language might have felt relatively higher levels of loss for being the volunteers [56]. Therefore, the loss-aversion tendency in the native language group increased the possibility of cooperating.

However, unlike the result of Game 4, the differences between players’ choices for each language treatment in Game 3 were not significant statistically. Inconsistent patterns in these two conditions of Volunteer’s Dilemmas may have been due to the probability judgements. Probability judgement is the essential component in making decisions about events with uncertainty. Decision makers mostly start from generating their understandings about the whole event, and then estimating the possibilities of alternative pathways before coming up with their strategies [57]. In these two cases, compared with the plane crash in Game 4, power failures might happen more frequently and a lot of the participants can experience these events directly. According to Volk et al. [11], situations which are easy to remember but occur rarely can result in greater levels of heuristic bias caused by foreign language effects. This could explain the larger differences between participants’ choices from each language treatment in Game 4.

Conclusively, assumptions about the foreign language effects on human decision making are still immature and the underlying mechanisms are unclear [58]. Therefore, to further evaluate the rationality of the Cultural Accommodation theory, or the underlying psychological mechanism governing how foreign languages impact on our daily decisions, experiments are being designed in our laboratory to test the foreign language effects of pairs of languages from the same cultural clusters (e.g., Chinese and Japanese; English and Dutch) or different cultural clusters (e.g., Chinese and English). Certainly, the nature of playing games involves the player’s cooperation and the goals. There are several factors affecting the processes: for example, the group size of the game, risk of the outcomes and individual involvement, etc. [59, 60]. These factors are all interacting with (or moderated by) the language effects (native and foreign) during decision making, and hence this is another important issue in dire need of investigation in future research. It is hoped that a more comprehensive picture of this topic can be revealed through solid, scientific evidence.

## Supporting information

S1 Appendix Games used in the study. (DOCX)
