# Supplementary material for: The foreign language effects on strategic behavior games
Source: PLoS One. 2022 Nov 17;17(11):e0277556. doi: 10.1371/journal.pone.0277556 (PMC9671371; doi:10.1371/journal.pone.0277556)
Supplement: S1 Appendix — (DOCX) [file pone.0277556.s001.docx]

Appendix: Games used in the study

**ENGLISH VERSION**

**Game 1**

Two suspects were under arrest for burglary, but the evidence available was not sufficient to prosecute them. The police interrogated the suspects detained in two separate rooms, each of them was in solitary confinement, without access to communication with the other.

The police told them, "If you and he(she) betray each other, each of you will serve one year in prison. If you betray him(her) but he(she) remains silent, you will be set free and he (she) will serve eight years in prison (and vice versa). If you and he(she) both remain silent, both of you will serve only six years in prison."

If you were one of the suspects, what would you do?

A. Confess

B. Not to confess

**Game 2**

Company A and company B are two competing companies in the same field.

If neither of them advertises their products, both of their profits will be 8 million dollars.

If one of them does not advertise but the other does, the firm which chooses to advertise will receive $10 million in revenue, thanks to increasing popularity, while the other one without advertising will lose a lot of consumers and only earn 2 million dollars.

However, if both companies advertise, not their revenue but their advertising cost will be increased, so the profits of both sides will be reduced to 4 million dollars.

If you were to make a decision on behalf of one of the companies, would you advertise without knowing the decision of your competitors?

A. To advertise

B. Not to advertise

**Game 3**

There was a sudden blackout in your community.

Every resident knew that as long as one person chose to call the power company (and pay for the call), the power company would restore the electricity supply for the entire community.

Will you make the call on your own or wait for someone else to call the power company?

A. Make the call by yourself

B. Wait for someone else to call the company

**Game 4**

You were traveling by plane with a group of strangers. The plane suddenly landed in a desert land due to an emergency. Although passengers were safe, you were all stuck in the communication blackout.

Everyone was discussing how to escape from this island. Soon some of you realized that the island was not so far away from the nearby, but a volunteer was needed to carry the SOS message to people outside the land. If not, you guys would all die on this desert island.

However, the only question was that there were a lot of sharks in the nearby waters. The danger would always be accompanied with this voluntary swimmer.

Assume that all of you could swim, would you be the voluntary swimmer?

A. Yes

B. No

**遊戲1**

警察逮捕了兩名竊盜罪嫌犯，然而，罪證不足以起訴兩名嫌疑人。警察將兩位嫌犯分別關在不同的牢房裡進行審問，他們無法彼此交流。

警察分別告訴兩人:「如果你們緘口不言，你們將共同面臨一年的牢獄；如果你背叛了他(她)選擇認罪坦白，你將被即時釋放然而他(她)將坐牢八年，反之亦然；如果你們都認罪，那麼你們將每人服刑六年。」

如果你是其中一名嫌犯，你會怎麼做?

A.坦白認罪

B.不坦白認罪

**遊戲2**

有兩家公司A和B, 彼此是競爭關係。

如果兩家都不登廣告，則它們的利潤分別為800萬。

倘若其中一家登上電視廣告，而另一家沒有這樣做，登廣告的品牌將因為知名度大增，扣除廣告成本還能賺到1000萬，沒登廣告的公司損失不少消費者，只能賺得200萬。

然而，若兩家都登廣告，則營收沒有增加，反而提高了廣告成本，因此雙方的利潤都降到了400萬元。

如果你要代表其中一間公司做出決策，在不知道競爭對手決策的情況下，你會選擇刊登廣告嗎?

A.刊登廣告

B.不刊登廣告

**遊戲3**

你所在的社區突然停電了。

包括你在內的所有社區居民都知道，只要有一個人選擇給供電公司撥打電話(並承擔通話費用) ，電力公司就會修復整個社區的電力供應。

你會選擇主動打電話給電力公司，還是等待別人打電話給電力公司?

A.主動打電話給電力公司

B.等待別人打電話

**遊戲4**

你和一群互不相識的人乘坐飛機旅行，途經一處荒島飛機出事故迫降了，一切通訊設備都在迫降中損壞，乘客都安然無恙。

所有人聚集在一起討論如何逃生。很快有人發現，這個小島離附近的大陸其實不遠，游泳可以到達大陸，從而找到人求救。如果沒有人遊到大陸傳遞求救資訊，那麼大家都會餓死在這個荒島上。

唯一的問題是，這附近的海域鯊魚很多，安全地遊過去需要一定的運氣。

假設大家都會游泳，你是否充當這個志願者，冒著風險遊過去解救大家?

A.我願意

B.我不願意
